# Telemedizinische Versorgung von Patienten mit kardialen Implantaten: Einblick in die telekardiologische Versorgungsrealität in Deutschland im Jahr 2022

**DOI:** 10.1007/s00399-024-01020-2

**Published:** 2024-05-24

**Authors:** Bianca Steiner, Bettina Zippel-Schultz, Erna Thoden, Christoph J. Geller, Thomas Klingenheben, Annett Kröttinger, Volker G. Leonhardt, Jens Placke, Thomas M. Helms

**Affiliations:** 1 Deutsche Stiftung für chronisch Kranke, Berlin, Deutschland; 2 Abteilung für Rhythmologie und invasive Elektrophysiologie, Zentralklinik Bad Berka GmbH, Bad Berka, Deutschland; 3 Praxis für Kardiologie Bonn, Im Mühlenbach 2b, 53127 Bonn, Deutschland; 4 Praxis Bad Wiessee, Bad Wiessee, Deutschland; 5 HIZ Berlin-MVZ GmbH, Berlin, Deutschland; 6 Gemeinschaftspraxis für Kardiologie, Rostock, Deutschland; 7 Peri Cor Arbeitsgruppe Kardiologie/Ass. UCSF, Hamburg, Deutschland

**Keywords:** Kardiologie, Telemedizin, Qualität der Gesundheitsversorgung, Geräte für die kardiale Resynchronisationstherapie, Implantierbare Defibrillatoren, Cardiology, Telecardiology, Quality of health care, Cardiac resynchronization therapy devices, Implantable defibrillators

## Abstract

**Hintergrund:**

Durch die Weiterentwicklung telemedizinischer Strukturen im deutschen Gesundheitswesen gewinnt das telekardiologische Monitoring zunehmend an Bedeutung, um eine ambulante, lückenlose und bedarfsgerechte Versorgung zu gewährleisten. Anhand der nationalen Qualitätssicherungsmaßnahme „DOQUVIDE – Dokumentation der Qualität bei der Erhebung von Vitalparametern durch implantierte Devices“ wird ein Ausschnitt der Versorgungsrealität von Patient*innen mit telekardiologischen Aggregaten in Deutschland abgebildet.

**Methodik:**

DOQUVIDE ist ein Messinstrument zur Erfassung der Versorgungsrealität ambulant telekardiologisch betreuter Patient*innen mit implantierten Schrittmacher‑/ICD-/CRT-P-/CRT-D-Devices und Ereignisrekordern. DOQUVIDE erfasst kardiale Ereignisse, telemedizinisch gewonnene Vitalparameter und das Prozedere nach Ereignismeldung.

**Ergebnisse:**

In 74 Praxen/Kliniken in 14 Bundesländern wurden im Jahr 2022 6678 Patient*innen telemedizinisch betreut; 937 wurden neu eingeschlossen. Diese waren durchschnittlich 77,8 Jahre alt mit mehrheitlich NYHA-Klasse II (62,6 %). Es wurden 5801 Ereignisbögen als Folge telekardiologischer Ereignisse generiert, davon 3590 aufgrund einer pathologischen Vorhofflimmerlast, 1812 aufgrund ventrikulärer Hochfrequenzepisoden, 295 durch Ereignisrekorder und 95 durch ausgelöste Gerätetherapien. Als Maßnahmen folgten vor allem telefonische Kontakte oder die ambulante Einbestellung der Patient*innen.

**Schlussfolgerung:**

Telekardiologisches Monitoring ist in der deutschen Versorgungsrealität angekommen. Standardisierte Prozesse und Qualitätssicherungsmaßnahmen ermöglichen die Etablierung gemeinsamer Qualitätsstandards sowie die Identifizierung von Weiterentwicklungspotenzialen und erleichtern den Praxen die Umsetzung im Versorgungsalltag.

## Abstract (en)

**Background:**

As telemedical structures continues to be developed in the German healthcare system, remote monitoring is becoming increasingly important to ensure comprehensive, outpatient, and tailored care. The national quality assurance measure “DOQUVIDE—Documentation of quality in the assessment of vital parameters by implanted devices” is used to provide insight into everyday care for patients with telemedicine-enabled devices in Germany.

**Methods:**

DOQUVIDE is a measuring instrument for recording outpatient remote monitoring for patients with implanted pacemaker, implantable cardioverter defibrillator (ICD), cardiac resynchronization therapy with a pacemaker (CRT-P), CRT defibrillator (CRT-D) devices and event recorder. DOQUVIDE records telemedically measured vital signs as well as cardiac events and the associated procedures initiated by cardiologists using standardized forms.

**Results:**

In 74 practices/clinics in 14 federal states, 6687 patients received telemedical care in 2022; 937 were newly enrolled. These (60% male) were on average 77.8 years old, mainly with New York Heart Association (NYHA) class II (62.6%). A total of 5801 electronic records were generated as a result of telecardiology events, of which 3590 were due to pathological atrial fibrillation and 1812 due to ventricular high-frequency episodes. 295 events were triggered by event recorders and 95 by device therapies. The main measures taken were telephone contacts or outpatient visits.

**Conclusion:**

Remote monitoring has become a reality in German healthcare. Standardized processes and the establishment of quality assurance measures enable the definition of common quality standards and the identification of the potential for further development and simplify implementation in day-to-day care for practices.

Kardiale Implantate sind ein wesentlicher Bestandteil der Diagnostik und Therapie symptomatischer Herzerkrankungen. Durch die Weiterentwicklung telemedizinischer Strukturen im deutschen Gesundheitswesen gewinnen sie immer mehr an Bedeutung, um eine ambulante, lückenlose und bedarfsgerechte Versorgung zu gewährleisten. Kardiologische Ereignisse, wie ventrikuläre Hochfrequenzepisoden, Vorhofflimmern und Bradykardien, können in Abhängigkeit des Aggregats telemedizinisch gemeldet werden. Aktuell fehlen jedoch adäquate Qualitätssicherungsmaßnahmen zum Prozedere nach Ereignismeldung.

## Hintergrund und Fragestellung

Kardiovaskuläre Erkrankungen (CVD), wie Vorhofflimmern, Herzinsuffizienz oder Herzinfarkt, sind mit über 340.000 Sterbefällen im Jahr 2021 eine der führenden Todesursachen in Deutschland [2]. Sie zählen mit einem Anteil von 20 % neben Krebs (20 %), Erkrankungen des Bewegungsapparats (11,4 %) sowie psychischen und neurologischen Störungen (8,5 %) zu den häufigsten chronischen Erkrankungen [8]. Zwar haben sich die Morbidität und Mortalität von kardiovaskulären Erkrankungen dank neuer Diagnose- und Therapieverfahren verbessert, doch bedingen der demografische Wandel und die damit einhergehende Alterung der Bevölkerung einen erneuten Anstieg der Prävalenz [2]. Kardiovaskuläre Erkrankungen stellen nicht nur für die betroffenen Patient*innen und ihre Angehörigen, sondern auch für das deutsche Gesundheitssystem eine erhebliche Belastung dar [3]. Ein effektives Monitoring von Patient*innen mit kardiovaskulären Erkrankungen gewinnt daher immer mehr an Bedeutung, um kardiale Dekompensationen frühzeitig zu erkennen, zeitnah interventionelle Maßnahmen einzuleiten, stationäre Behandlungen zu verhindern, die Lebensqualität der Betroffenen zu verbessern und gleichzeitig das Gesundheitssystem zu entlasten [1, 9, 11].

Dabei nehmen kardiale Aggregate, wie implantierte Herzschrittmacher (HSM), Defibrillatoren (ICD) und Systeme zur kardialen Resynchronisationstherapie (CRT), eine entscheidende Rolle ein [12]. Moderne kardiale Aggregate übertragen als telemedizinische Geräte drahtlos und ohne Arzt-Patienten-Kontakt verschiedene technische und klinisch relevante Parameter, wie die mittlere ventrikuläre Frequenz, Vorhofflimmer-Last (AF-Burden), Zahl der Therapien mittels antitachykardem Pacing (ATP) bzw. Schockabgaben (ICD/CRT-D) oder die Zahl der ineffektiven/inappropriaten/unnötigen Schocks (ICD/CRT‑D; [4]). Ebenso können kardiologische Ereignisse, wie ventrikuläre Hochfrequenzepisoden und Bradykardien, in Abhängigkeit des Aggregats, telemedizinisch gemeldet werden. Je nach Hersteller ist es außerdem möglich, zusätzliche Alarme zu implementieren, bspw. bei Überschreitung bestimmter Schwellenwerte [4]. Die für die Implantation der Aggregate erforderlichen kardiologischen Eingriffe, darunter Schrittmacherimplantationen und -wechsel, können zunehmend ambulant durchgeführt werden [15]. Diese Ambulantisierung ist integraler Teil eines umfassenden Wandels im deutschen Gesundheitswesen. Auch die Weiterentwicklung telemedizinischer Strukturen auf technischer, organisatorischer und politischer Ebene fördert die Ambulantisierung mit dem Ziel, eine lückenlose und bedarfsgerechte Versorgung kardiovaskulärer Erkrankungen zu gewährleisten. Dabei legen der Beschluss „Telemonitoring Herzinsuffizienz“ des Gemeinsamen Bundesausschusses (G-BA) sowie die „Qualitätssicherungsvereinbarung Telemonitoring bei Herzinsuffizienz“ bereits grundlegende Qualitätskriterien fest [5, 10]. Jedoch ist eine weiterführende Qualitätssicherung (QS) erforderlich, um zu gewährleisten, dass die aus kardialen Aggregaten gewonnen Erkenntnisse tatsächlich effektiv in die klinische Praxis integriert werden. Die QS-Maßnahme „DOQUVIDE – Dokumentation der Qualität bei der Erhebung von Vitalparametern durch implantierte Devices“ soll daher Einblicke in die Praxis der telekardiologischen Versorgung in Deutschland im Jahr 2022 geben und Handlungsbedarfe im Bereich der Qualitätssicherung praxisnah aufzeigen.

## Methodik

### Messinstrument und Dokumentation von Maßnahmen

DOQUVIDE ist ein Messinstrument zur Erfassung der Versorgungsrealität von Patient*innen mit implantierten Schrittmacher‑/ICD-/CRT-P/CRT-D-Devices und Ereignisrekordern. Ergänzend zu kardialen Ereignissen und Vitalparametern, die telemedizinisch von Patient*innen mit telekardiologischen Aggregaten gewonnen werden, erfasst DOQUVIDE das diagnostische und therapeutische Prozedere nach Ereignismeldung. Hierzu werden vier selbstentwickelte, standardisierte Formulare, sogenannte Ereignisbögen, verwendet. Welcher Ereignisbogen vom überwachenden Arzt bzw. qualifizierten Mitarbeiter oder der überwachenden Ärztin bzw. qualifizierten Mitarbeiterin im elektronischen Dokumentationssystem auszufüllen ist, hängt im Wesentlichen vom zugrundeliegenden kardialen Ereignis ab:

- AF-Burden: auszufüllen bei telemedizinischer Meldung einer pathologischen Vorhofflimmerlast
- ATP-Schock: auszufüllen bei telemedizinischer Meldung einer stattgefundenen Gerätetherapie (ATP oder Schock)
- Ereignisrekorder: auszufüllen bei telemedizinischer Meldung eines durch einen implantierten Ereignisrekorder erfassten Ereignisses, z. B. Bradykardie, Tachykardie oder neu aufgetretenes Vorhofflimmern
- HVF: auszufüllen bei telemedizinischer Meldung von ventrikulären Hochfrequenzepisoden bei Herzschrittmacherpatient*innen

Nach Meldung eines kardialen Ereignisses durch ein Aggregat wird vom elektronischen Dokumentationssystem automatisch der zugehörige Ereignisbogen generiert und eine entsprechende Aufgabe im System hinterlegt. Verschiedene Validierungsstatus ermöglichen es, Ereignisbögen initial auszufüllen, zu validieren und schließlich für die QS und Auswertung freizugeben. Die an DOQUVIDE teilnehmenden Praxen/Kliniken erhalten quartalsweise und jährlich Berichte zur Überprüfung der Dokumentationsqualität.

### Datenanalyse

Die Auswertung der Daten erfolgte in erster Linie für die im Jahr 2022 neu ins QS-gesicherte kardiale Telemonitoring mittels DOQUVIDE eingeschlossenen Patient*innen. Sofern zeitliche Unterschiede von Relevanz waren, wurden die letzten drei Jahre zum Vergleich herangezogen. Die statistische Analyse umfasst gängige Methoden der deskriptiven Statistik sowie Subgruppenanalysen nach Regionen.

## Ergebnisse

### Teilnehmende Praxen

**Abb. 1:**
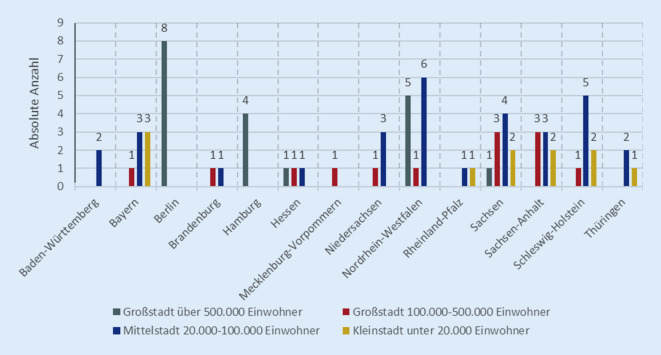
Standort der Praxen im Jahr 2022 nach Bundesland und Städtegröße

Insgesamt haben im Jahr 2022 74 Praxen/Kliniken am QS-gesicherten kardialen Telemonitoring mittels DOQUVIDE teilgenommen, vor allem aus Nordrhein-Westfalen und Sachsen (Abb. 1). Aus Bremen und dem Saarland liegen keine Daten vor. Die Anzahl der teilnehmenden Praxen ist in den letzten drei Jahren stabil geblieben (2023: *n* = 74; 2021: *n* = 75).

Im Jahr 2022 wurden 937 Patient*innen neu zum Telemonitoring unter Begleitung der QS-Maßnahme DOQUVIDE eingeschlossen. Durchschnittlich waren es 14 Patient*innen pro Praxis, wobei die Streuung mit 17,4 Patient*innen hoch ist. Knapp 60 % der Praxen schlossen weniger als 10 Patient*innen. 28,4 % dieser Praxen nahmen zwar an DOQUVIDE teil, schlossen aber aktiv keine Patient*innen ein. Lediglich sechs Praxen (8 %) schlossen mehr als 40 Patient*innen ein. Davon sind vier in den Großstädten Hamburg, Dortmund, Chemnitz und Essen ansässig, eine in der Mittelstadt Rendsburg (Schleswig-Holstein) und eine in der Kleinstadt Diez (Rheinland-Pfalz). Es ließen sich keine regionalen Unterschiede in der Anzahl der eingeschlossenen Patient*innen nach Städtegröße feststellen.

Insgesamt wurden 6687 Patient*innen aktiv unter Begleitung von DOQUVIDE mittels Monitoring überwacht. Im Durchschnitt waren es 90 Patient*innen pro Praxis (SD = 117,41). 22 % der Praxen überwachten weniger als 10 Patient*innen; 8 % sogar keine. Vierzehn Praxen (20 %) überwachten jährlich mehr als 200 Patient*innen, zwei davon sogar mehr als 400. Beide Praxen befinden sich in Großstädten mit über 500.000 Einwohner (Berlin, Hamburg).

### Durchgeführte Implantationen

Im Jahr 2022 wurden insgesamt 937 Implantationen registriert, rund 14 % weniger als im Vorjahr; trotz gleichbleibender Anzahl teilnehmender Praxen. Mögliche Ursachen hierfür könnten der G‑BA-Beschluss zum Telemonitoring bei Herzinsuffizienz und die zugehörige QS-Vereinbarung sein. Diese schließen seit August 2021 Patient*innen mit fortgeschrittener Herzinsuffizienz (NYHA II oder III) und reduzierter kardialer Pumpleistung (Ejektionsfraktion/EF < 40) je nach gewähltem Monitoring weitgehend von einer Teilnahme an DOQUVIDE aus. Im Vergleich dazu zeigen sich in den Vorjahren nur geringe Unterschiede (2021: *n* = 1086; 2020 = 987).

#### Implantationsgrund

Häufigste Indikation für die Implantation eines kardiologischen Implantats war das Sick-Sinus-Syndrom (SSS, I49.5), gefolgt vom atrioventrikulären Block (AV-Block, I44) in unterschiedlichen Schweregraden. Unter SSS werden verschiedene Formen zusammengefasst: SSS ohne Angaben (*n* = 220), SSS Brady-Tachykardie (*n* = 100), SSS + AV-Block (*n* = 47), SSS Sinuatrialer Arrest (*n* = 21) und SSS SA-Exit-Block (*n* = 9).

Neben der pharmakologischen Therapie zählt die Implantation eines HSM sowohl beim SSS, als auch beim AV-Block zur Standardtherapie [7]. Während beim AV-Block die ventrikuläre Erregung sichergestellt werden soll, unterstützt oder übernimmt der HSM beim SSS die Aufgaben des Sinusknotens [6, 7]. Dementsprechend wurden beim SSS in der Regel HSM neu implantiert (48,1 %) oder gewechselt (51,1 %). Gleiches galt für den AV-Block 1. und 2. Grades. Nur selten (*n* = 2; 0,8 %) wurde beim AV-Block 3. Grades (QRS ohne Angaben) von einer HSM-Therapie abgewichen und auf einen ICD oder CRT upgegradet. Diese Patient*innen wiesen NYHA-Klasse II auf.

Entsprechend der zuvor beschriebenen Indikationsverteilung machten Neuimplantationen (44,8 %) und Wechsel (50,6 %) von HSM zusammen mehr als 90 % der durchgeführten Implantationen aus. Neuimplantationen von ICD und Ereignisrekordern, der Wechsel von CRT sowie das Upgrading von HSM auf ICD/CRT machten nur einen marginalen Anteil von jeweils weniger als 1 % aus. Der Wechsel von ICDs (2 %) ist ebenfalls selten. Insgesamt waren Gerätewechsel mit 53,6 % deutlich häufiger als Neuimplantation (44,8 %).

**Abb. 2:**
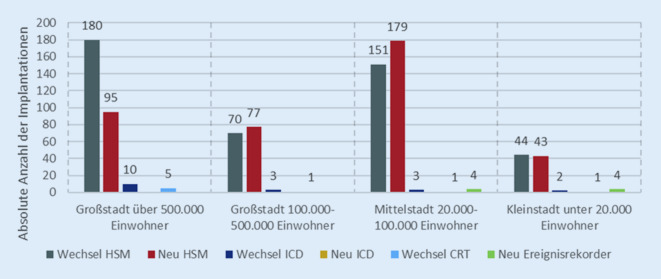
Anteil der Implantationen nach Region; Gesamtheit aller DOQUVIDE-Patient*innen

In Großstädten mit mehr als 500.000 Einwohnern fanden überwiegend Gerätewechsel statt, insbesondere von HSM (Abb. 2). Mit 62,1 % aller Eingriffe war der Anteil der HSM-Wechsel deutlich größer als in den anderen Regionen. Neuimplantationen von HSM fanden mit 32,8 % hingegen deutlich seltener statt. Es zeigte sich außerdem, dass Wechsel von ICD (55,6 %) und CRT (62,5 %) überwiegend in Großstädten mit mehr als 500.000 Einwohnern durchgeführt wurden.

### Eingeschlossene Patient*innen

In den letzten drei Jahren ist das Geschlechterverhältnis der neu in DOQUVIDE registrierten Patient*innen weitgehend stabil geblieben. Rund 60 % der Betroffenen sind männlich. Dies entspricht dem generellen Geschlechterverhältnis von Herzerkrankungen in Deutschland. Laut dem 34. Deutschen Herzbericht entfielen im Jahr 2021 etwa 58 % der ausgewählten kardiologischen Diagnosen^1^ (vollstationäre Fälle) auf Männer und 42 % auf Frauen [2].

Der Großteil der kardiologischen Patient*innen in Deutschland gehört der Altersgruppe der über 65-Jährigen an. Die höchsten Erkrankungsraten gibt es für ischämische Herzkrankheit und Herzrhythmusstörungen bei Männern im Alter von 75 bis 80 Jahren und bei Frauen im Alter zwischen 80 und 85 Jahren, für Herzklappenerkrankungen in der Altersgruppe der 85- bis 90-Jährigen und für Herzinsuffizienz in der Altersgruppe über 90 Jahre [2]. Im Jahr 2022 lag das Durchschnittsalter der neu in DOQUVIDE eingeschlossenen Patient*innen bei 77,8 Jahre (± 10,5). Etwa die Hälfte der Patient*innen war über 80 Jahre alt. Im Durchschnittsalter zeigten sich geringe Geschlechtsunterschiede, wobei Frauen im Schnitt 79 Jahre (± 9,1) und Männer 76,7 Jahre (± 11,6) alt waren. 58 % der erkrankten Frauen waren über 80 Jahre alt, 10 % sogar über 90 Jahre. Dagegen fielen *nur* etwa 50 % der Männer in die Altersklasse der über 80-Jährigen und nur etwa 5 % in die der über 90-Jährigen. Dies lässt sich u. a. durch die höhere Lebenserwartung von Frauen sowie die altersabhängige Morbidität von Herzerkrankungen bei Frauen und Männern erklären [2].

#### Schweregrad der Erkrankung

Vorhofflimmern ist eine der häufigsten Herzrhythmusstörungen in Deutschland, von der etwa 2 % der Bevölkerung betroffen sind [2]. Etwa 9–16 % der über 80-Jährigen sind betroffen [14]. Rund die Hälfte der im Jahr 2022 neu eingeschlossenen Patient*innen hatte ein dokumentiertes Vorhofflimmern, wobei es keine signifikanten Geschlechtsunterschiede gab (*p* = 0,455).

Die meisten Patient*innen in Deutschland haben eine normale Ejektionsfraktion (EF > 50 %). Mittelgradige (EF = 30–40 %) bis schwere Einschränkungen (EF < 30 %) der Pumpfunktion sind selten (Ponikowski et al., 2016). Dies spiegelte sich auch in den DOQUVIDE-Daten aus 2022 wider. 7 % der Patient*innen wiesen eine leichtgradig eingeschränkte Pumpleistung auf (EF = 40–49 %). Patient*innen mit mäßigen Einschränkungen litten häufig an einem SSS (SSS + AV-Block, SSS Brady-Tachykardie, SSS ohne Angabe jeweils 13,3 %) oder an ventrikulären Tachykardien. Die Altersanalyse zeigte, dass der Schweregrad der Erkrankung mit zunehmendem Alter bis 69 Jahre leicht zunahm (Tab. 1). Auch zwischen den Geschlechtern zeigten sich signifikante Unterschiede (*p* = 0,0091). Männer (11,89 %) litten signifikant häufiger an Einschränkungen der Pumpleistung des Herzens als Frauen (6,03 %).

**Tab. 1:** Schweregrad der Erkrankungen nach Altersgruppen im Jahr 2022 nach Ejektionsfraktion (EF)

Altersklasse (Jahre)	Normal (%)	Leichtgradig (%)	Mittelgradig (%)	Hochgradig (%)	Keine Angabe (%)
18–49	90,9	0,0	0,0	0,0	9,1
50–59	82,8	3,4	3,4	0,0	10,3
60–69	81,6	11,7	2,9	0,0	3,9
70–79	85,3	4,9	1,5	0,0	8,3
> 80	83,1	7,7	1,5	0,2	7,5

Die Mehrheit der Patient*innen wies die NYHA-Klasse II auf (62,6 %), was auf ein mittleres Erkrankungsstadium hindeutet. Nur wenige Patient*innen fielen in die NYHA-Klasse III, keine in NYHA-Klasse IV. Patient*innen der NYHA-Klasse III litten häufig an einem SSS oder einem AV-Block 3. Grades. Altersunterschiede in Bezug auf die NYHA-Klasse waren erkennbar, wobei ältere Patient*innen tendenziell einen höheren Schweregrad aufwiesen. Während jüngere Patient*innen zwischen 18 und 49 Jahren in 36,4 % der Fälle einen geringen Schweregrad der Erkrankung (NYHA-Klasse I) aufwiesen, sind es in der Altersklasse der 50- bis 59-Jährigen nur 20,7 % und in der Altersklasse der über 80-Jährigen nur noch 10,3 %. Es lagen keine signifikanten Geschlechtsunterschiede vor (*p* = 0,28). Sowohl Männer (63,5 %) als auch Frauen (61,4 %) waren am häufigsten in die NYHA-Klasse II einzugruppieren.

#### Regionale Unterschiede

Es zeigte sich, dass der Anteil der Patient*innen mit höherem Schweregrad (NYHA II und III) in Hessen, Sachsen, Brandenburg, Berlin und Schleswig-Holstein besonders hoch war. Dies lässt sich u. a. durch die Bevölkerungsanteile in den Bundesländern erklären. So ist der Anteil der über 65-Jährigen mit einem erhöhten Risiko für eine kardiovaskuläre Erkrankung in diesen Bundesländern höher als im übrigen Bundesgebiet [2]. Darüber hinaus gibt es Versorgungsunterschiede zwischen den Bundesländern und Regionen (urban vs. ländlich), die u. a. zu höheren Sterblichkeitsraten in Sachsen-Anhalt und in einigen ostdeutschen Landkreisen führen [13]. Die DOQUVIDE-Daten zeigten einen signifikanten Unterschied im Schweregrad der Erkrankung nach NYHA zwischen alten und neuen Bundesländern (*p* = 0,044). So war der Anteil der Patient*innen in der NYHA-Klasse I in den neuen Bundesländern mit 27,1 % vergleichsweise hoch, ebenso aber auch der Anteil der Patient*innen mit NYHA III.

#### Komorbiditäten

Zusätzlich zu ihrer kardiologischen Erkrankung litten 277 der 937 Patient*innen, die 2022 neu in DOQUVIDE eingeschlossen wurden, an mindestens einer weiteren Erkrankung bzw. Beschwerden, die eine medikamentöse Behandlung erforderten. Zu den häufigsten Komorbiditäten gehörten Diabetes mellitus (12,1 %), Schilddrüsenerkrankungen (5,8 %) und gastroösophageale Beschwerden (8,5 %), Depressionen einschließlich Angst- und Schlafstörungen sowie Wahnvorstellungen (5 %).

### Pharmakologische Therapie

**Abb. 3:**
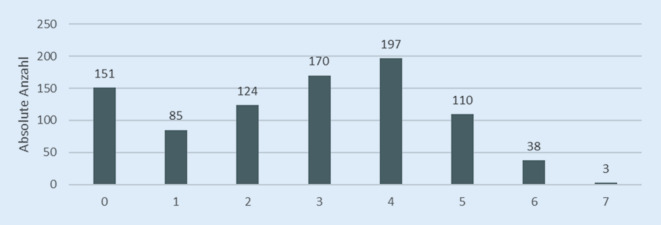
Anzahl der eingenommenen kardialen Medikamente der im Jahr 2022 neu eingeschlossenen Patient*innen

Im Jahr 2022 nahmen 83 % der neu in DOQUVIDE eingeschlossenen Patient*innen bei Einschluss mindestens ein kardiales Medikament ein. Bei den übrigen Patient*innen wurde keine kardiale Medikation erfasst (Abb. 3). Nur bei weniger als 1 % wurde explizit angegeben, dass keine kardiale Medikation eingenommen wird. Die Mehrheit der Patient*innen nahm mehr als ein kardiales Medikament ein, im Durschnitt 2,77 (± 1,79). Die Anzahl der eingenommenen kardialen Medikamente stieg mit dem Alter. Betablocker und/oder Antikoagulanzien wurden bei Einschluss von 17 % der Patient*innen am häufigsten dokumentiert.

Etwa ein Drittel der im Jahr 2022 neu eingeschlossenen Patient*innen (32 %) nahm bei Einschluss in DOQUVIDE auch nichtkardiale Medikamente ein. Die meisten (57 %) nahmen ein weiteres nichtkardiales Medikament ein; im Durchschnitt sind es 0,7 (± 1,38). Es ist jedoch zu beachten, dass bei etwa der Hälfte dieser 158 Patient*innen (*n* = 76) nur die Information „Andere Medikamente“ angegeben wurde, ohne weitere spezifizierende Einzelheiten. Somit könnten die tatsächliche Anzahl der eingenommenen nichtkardialen Medikamente pro Patient*in sowie die durchschnittliche Anzahl der zusätzlich eingenommenen Medikamente real (deutlich) höher sein als hier berichtet. Am häufigsten wurden nichtopioide Analgetika (13,6 %), Blutverdünner (12,2 %) und Diabetesmedikamente (12,1 %) eingenommen.

### Ereignisse, Ereignisbögen und eingeleitete Maßnahmen

**Abb. 4:**
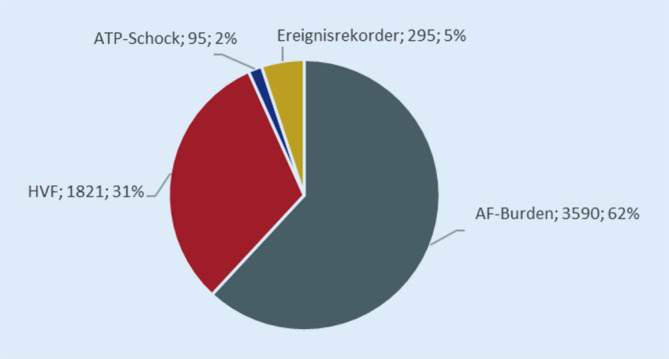
Anzahl der im Jahr 2022 generierten und von den Praxen/Kliniken bearbeiteten Ereignisbögen

Für die 6687 im Jahr 2022 aktiv im QS-gesicherten kardialen Telemonitoring mittels DOQUVIDE betreuten Patient*innen wurden 5801 Ereignisbögen als Folge telekardiologischer Ereignisse generiert und von den Praxen bearbeitet (Abb. 4).

Auf fast die Hälfte (45 %) der 3590 gemeldeten AF-Burden-Ereignisse folgten diagnostische oder therapeutische Maßnahmen. Am häufigsten wurden die Patient*innen angerufen (64,4 %) oder ambulant einbestellt (22,7 %). Medikationsanpassungen fanden nur in 45 Fällen (2,8 %) statt.

Bei 60,7 % der 1812 gemeldeten HVF-Ereignisse wurden diagnostische oder therapeutische Maßnahmen eingeleitet. In drei Viertel der Fälle wurden die Patient*innen angerufen. Invasive oder nichtinvasive diagnostische Maßnahmen wurden nur selten veranlasst. Die Medikation wurde nur in 25 Fällen (2,3 %) angepasst.

Ereignisrekorder meldeten insgesamt 295 Ereignisse. Neu aufgetretenes Vorhofflimmern (34,2 %), Bradykardien < 50 Schläge/min (21 %) und Tachykardien > 100 Schläge/min (20,3 %) waren die häufigsten Ursachen. Nach Ereignismeldung wurden die Patient*innen in 30,8 % der Fälle kontaktiert, hauptsächlich telefonisch (84,6 %). Ambulante Einbestellungen erfolgten in 12,1 %, stationäre Einweisungen nur in 3,3 % der Fälle. Therapeutische oder diagnostische Maßnahmen wurden selten eingeleitet (15,9 %). Am häufigsten kamen hierbei nichtinvasive diagnostische Verfahren zum Einsatz. Implantat-gestützte Therapien folgten nur in 6,4 % (*n* = 5) der Fälle. In der Regel wurde ein 2‑Kammer-Herzschrittmacher implantiert (*n* = 4), in einem einzigen Fall ein 2‑Kammer-ICD.

Die 95 gemeldeten Gerätetherapien (ATP oder Schock) erfolgten am häufigsten in Ruhe (56,4 %) oder während einer symptomatischen Therapie (30,8 %). Die vom Gerät gestellte Diagnose und die ausgelöste Therapie (ATP oder Schock) wurden von den überwachenden Ärzt*innen im Allgemeinen als korrekt bzw. erfolgreich eingestuft. In fast 10 % der Fälle war die Gerätetherapie nicht angemessen oder unnötig. Bei zwei Patient*innen war die Gerätetherapie nicht erfolgreich, davon lag in einem Fall ein Elektrodenfehler vor. Auf jedes gemeldete ATP/Schock-Ereignis folgte eine Maßnahme. In den meisten Fällen wurden die Patient*innen angerufen (53,7 %) oder ambulant einbestellt (27,4 %). Die kardiale Medikation wurde in 5,2 % der Fälle angepasst.

## Diskussion

Die Patientencharakteristika der 937 im Jahr 2022 neu in DOQUVIDE eingeschlossenen Patient*innen geben einen umfassenden Einblick in die demografischen Merkmale und den Schweregrad der kardiologischen Erkrankungen innerhalb der untersuchten Patientenkohorte. Verschiedene Ereignisbögen bilden das Spektrum des therapeutischen und diagnostischen Prozedere nach Ereignismeldung ab, darunter AF-Burden, ATP/Schock, HVF und Ereignisrekordermeldungen. In der Regel wurden die Patient*innen zunächst telefonisch kontaktiert oder ambulant einbestellt, um weiterführende Informationen über ihren Zustand und ihre Symptome vor, während und nach der Ereignismeldung zu erfassen. So konnten patientenindividuell gezielte weitere diagnostische und therapeutische Maßnahmen eingeleitet werden.

Bei der Interpretation der Ergebnisse sollte berücksichtigt werden, dass DOQUVIDE nur einen Ausschnitt der aktuellen gesamtdeutschen Versorgungsrealität betrachtet. So liegen bspw. aus Bremen und dem Saarland keine Daten und aufgrund der geringen Anzahl teilnehmender Praxen/Kliniken aus einigen anderen Bundesländern nur begrenzt Daten vor. Dies führt zu einem Selektionsbias durch die eingeschlossenen Praxen. Dementsprechend sind nur in einem beschränkten Umfang Aussagen zu regionalen Unterschieden möglich. Darüber hinaus beteiligen sich nur drei der fünf großen Gerätehersteller an DOQUVIDE. Aufgrund des überragenden Marktanteils dieser Hersteller in Deutschland im ambulanten Sektor ist eine etwaige Herstellerabhängigkeit der Ergebnisse jedoch als unproblematisch anzusehen.

Da kein elektronischer Medikationsplan in DOQUVIDE vorliegt und nicht alle Praxen die komplette Medikation der Patient*innen erfassten, weisen die Auswertungen der Medikation Schwächen auf, sodass die tatsächliche Medikamenteneinnahme unter Umständen unterschätzt wird. Die hohe Anzahl an Einträgen „Andere Medikamente“ erschwert die genaue Zuordnung und Bewertung der Begleitmedikation zusätzlich. Ebenfalls wird die retrospektive Ableitung von Komorbiditäten erschwert. Diese werden in den DOQUVIDE-Formularen nicht explizit erfasst, sondern lediglich aus der eingenommenen Medikation abgeleitet. Es ist daher davon auszugehen, dass der tatsächliche Anteil der Patient*innen mit einer oder mehreren Komorbiditäten deutlich höher ist als angegeben. Vertiefende Aussagen zu Komorbiditäten und entsprechende Subgruppenanalysen sind nicht möglich.

Trotz der genannten Limitationen leisten die vorliegenden Ergebnisse einen wesentlichen Beitrag zur Verbesserung der Telekardiologie in Deutschland. Indem sie die telekardiologische Versorgungsrealität detailliert analysieren, tragen sie zur Weiterentwicklung und unabhängigen Bewertung der Effektivität und Effizienz des ambulanten kardialen Telemonitorings bei (Qualitätssicherung). Auf politischer Ebene unterstützen die Ergebnisse die Entwicklung eines einheitlichen Qualitätsstandards, fördern die Transparenz der Versorgung und zeigen Effizienzpotenziale auf. Zukünftige Untersuchungen und Weiterentwicklungen der QS-Maßnahme DOQUVIDE sollten darauf abzielen, Daten noch systematischer zu erfassen, zu speichern und zu analysieren, um nicht nur die Qualität des ambulanten Telemonitorings zu bewerten, sondern auch Qualitätsindikatoren abzuleiten. Schließlich könnten somit Trends im kardialen Telemonitoring besser erfasst, Verbesserungen identifiziert und Weiterentwicklungspotenziale für medizinische Leitlinien und Praktiken abgeleitet werden.

## Fazit

Zusammenfassend lässt sich festhalten, dass telekardiologisches Monitoring in Deutschland aufgrund seiner Bedeutung für die effiziente Überwachung von Patienten mit kardiovaskulären Erkrankungen und der verbesserten Versorgung im Gesundheitswesen eine wichtige Rolle spielt. Die Integration von kardialen Aggregaten in diese Überwachungsstrategien ist dabei ein wesentlicher Bestandteil, um eine umfassende Betreuung und Früherkennung von Herzproblemen zu gewährleisten. Die telemedizinische Betreuung von Patient*innen mit kardialen Aggregaten und die Schaffung von QS-Maßnahmen sind Schlüsselfaktoren, um eine hochwertige und patientenzentrierte Versorgung sicherzustellen.

## Footnotes

1 Ischämischen Herzkrankheiten (I20–I25), akuter Myokardinfarkt (I21), Herzklappenkrankheiten (I05–I09, I34–I39), Herzrhythmusstörungen (I44–I49), Herzinsuffizienz (I50), angeborene Fehlbildungen des Kreislaufsystems (Q20–Q28).
